# Genetic ancestry, admixture and health determinants in Latin America

**DOI:** 10.1186/s12864-018-5195-7

**Published:** 2018-12-11

**Authors:** Emily T. Norris, Lu Wang, Andrew B. Conley, Lavanya Rishishwar, Leonardo Mariño-Ramírez, Augusto Valderrama-Aguirre, I. King Jordan

**Affiliations:** 1 School of Biological Sciences, Georgia Institute of Technology, Atlanta, GA 30332 USA; 2 PanAmerican Bioinformatics Institute, Cali, Valle del Cauca Colombia; 3 IHRC-Georgia Tech Applied Bioinformatics Laboratory (ABiL), Atlanta, GA USA; 4 National Center for Biotechnology Information, National Library of Medicine, National Institutes of Health, Bethesda, MD USA; 5 Biomedical Research Institute, Faculty of Health, Universidad Libre-Seccional Cali, Cali, Valle del Cauca Colombia

**Keywords:** Genetic ancestry, Admixture, Ancestry-enrichment, Adaptive introgression, Health, Disease, Population genetics, Immune system

## Abstract

**Background:**

Modern Latin American populations were formed via genetic admixture among ancestral source populations from Africa, the Americas and Europe. We are interested in studying how combinations of genetic ancestry in admixed Latin American populations may impact genomic determinants of health and disease. For this study, we characterized the impact of ancestry and admixture on genetic variants that underlie health- and disease-related phenotypes in population genomic samples from Colombia, Mexico, Peru, and Puerto Rico.

**Results:**

We analyzed a total of 347 admixed Latin American genomes along with 1102 putative ancestral source genomes from Africans, Europeans, and Native Americans. We characterized the genetic ancestry, relatedness, and admixture patterns for each of the admixed Latin American genomes, finding a spectrum of ancestry proportions within and between populations. We then identified single nucleotide polymorphisms (SNPs) with anomalous ancestry-enrichment patterns, i.e. SNPs that exist in any given Latin American population at a higher frequency than expected based on the population’s genetic ancestry profile. For this set of ancestry-enriched SNPs, we inspected their phenotypic impact on disease, metabolism, and the immune system. All four of the Latin American populations show ancestry-enrichment for a number of shared pathways, yielding evidence of similar selection pressures on these populations during their evolution. For example, all four populations show ancestry-enriched SNPs in multiple genes from immune system pathways, such as the cytokine receptor interaction, T cell receptor signaling, and antigen presentation pathways. We also found SNPs with excess African or European ancestry that are associated with ancestry-specific gene expression patterns and play crucial roles in the immune system and infectious disease responses. Genes from both the innate and adaptive immune system were found to be regulated by ancestry-enriched SNPs with population-specific regulatory effects.

**Conclusions:**

Ancestry-enriched SNPs in Latin American populations have a substantial effect on health- and disease-related phenotypes. The concordant impact observed for same phenotypes across populations points to a process of adaptive introgression, whereby ancestry-enriched SNPs with specific functional utility appear to have been retained in modern populations by virtue of their effects on health and fitness.

**Electronic supplementary material:**

The online version of this article (10.1186/s12864-018-5195-7) contains supplementary material, which is available to authorized users.

## Background

The modern human species – *Homo sapiens sapiens* – originated in sub-Saharan Africa ~ 200,000 years ago and began to migrate out of Africa and spread throughout the world starting ~ 70,000 years ago [1, 2]. After heading north out of Africa, humans spread to the east and west, populating Melanesia, Asia, and Europe, and eventually made their way across the Bering Strait into the Americas ~ 20,000 years ago. As human populations occupied different parts of the globe, they often became geographically isolated in their new homelands. Thousands of years of geographic isolation were accompanied by population genetic diversification, giving rise to the diverse human population groups that can be seen around the world to this day [3, 4]. Distinct continental population groups – African, Asian, and European in particular – are the most obvious examples of this evolutionary process. There were, of course, a number of episodes of genetic admixture during this time [5], whereby previously isolated populations came into contact and began to mix, but for the most part, the dominant mode of human evolution since our emergence from Africa has been characterized by populations’ physical isolation followed by genetic diversification.

This pattern of human evolution was turned upside down upon the arrival of Columbus in the New World a mere 500 years ago, which is less than 1% of the elapsed time since humans emerged from Africa. Columbus’ voyages precipitated the so-called ‘Columbian Exchange’ – a massive transfer of life forms, which had evolved separately for millennia, between the Old and New World hemispheres [6, 7]. The human dimension of the Columbian Exchange entailed genetic admixture between previously isolated populations on an unprecedented scale, in terms of both scope and rapidity [8]. The conquest and colonization of the Americas, along with the trans-Atlantic slave trade, brought African, European, and Native American populations into close and sustained contact for the first time. As a consequence, these diverse population groups began to mix, giving rise to novel admixed American populations. This is particularly true for Latin America, where populations are characterized by high levels of genetic admixture among African, European, and Native American ancestral source populations [9–11].

Latin American genomes can thus be considered to represent a recent innovation in human evolution. Indeed, genomes from modern Latin American populations are evolutionarily novel in the sense that they contain combinations of genetic variants (haplotypes) that never previously existed together on the same genetic background. Our group is interested in trying to understand the implications of the recent advent of novel Latin American genomes, particularly as it relates to the genetic determinants of health-related phenotypes. In other words, we are asking what it means when genomes that were separated for many thousands of years are suddenly brought back together and what the implications of this process are for human health and fitness.

Our group and others have employed an approach that we call ancestry-enrichment analysis to address these kinds of questions via population-level studies of admixed American genomes [8]. This approach relies on the characterization of local patterns of genetic ancestry for individual genomic loci. Local ancestry assignment, colloquially referred to as chromosome painting, entails the delineation of ancestral origins of specific haplotypes across the genome. The resulting chromosome paintings reveal the genomes of admixed individuals as mosaics of interspersed ancestry-specific haplotypes. When a population sample of admixed genomes is characterized in this way, the percent ancestry contributions from each ancestral source population can be computed for all haplotype loci genome-wide. Ancestry-enrichment analysis then entails the identification of specific haplotype loci that have anomalous patterns of local ancestry, i.e. levels of locus-specific ancestry that are significantly higher or lower than can be expected by chance given the overall ancestry profile of the population. Statistically significant signals of ancestry-enrichment are taken as evidence of adaptive introgression, whereby introgressed haplotypes increase in frequency by virtue of a selective advantage that they provide to individuals in an admixed population.

A number of recent studies have used ancestry-enrichment analysis to show evidence of adaptive introgression in admixed American genomes. The first study of this kind showed an excess of African ancestry at the major histocompatibility locus (MHC) in a sample of Puerto Rican genomes [12], and a follow up study several years later also found ancestry-enrichment at the same region in a Mexican population [13]. Since that time, several other studies have replicated the finding of ancestry-enrichment in admixed populations at this and other health related loci [14–18]. Our own more recent work on Colombian genome sequences revealed even more widespread ancestry-enrichment, which impacted various aspects of the immune system, including pathways involved in both innate and adaptive immunity [18].

All of the previous ancestry-enrichment studies were distinguished by their interrogation of the ancestral origins of individual haplotypes, i.e. physically linked sets of genetic variants that are inherited together. For this study, we developed and applied a novel method based on individual genetic variants – single nucleotide polymorphisms (SNPs) – in an effort to expand our view of the relationship between genetic ancestry, admixture and health in Latin American populations. Our approach entails the detection of SNPs that are found at anomalously high frequencies in admixed populations compared to what is expected based on their frequencies in the ancestral source populations, i.e. ancestry-enriched SNPs. To find such ancestry-enriched SNPs, we consider the proportional contributions of ancestral source populations to admixed Latin American populations, together with SNP frequencies in the ancestral populations, to derive expected SNP frequencies for the Latin American populations. These expected frequencies are compared to observed frequencies in order to identify statistically significant ancestry-enriched SNPs; the connection between ancestry-enriched SNPs and health-related phenotypes is then explored via analysis of the functional annotations of the SNPs and their linked genes. In particular, we interrogated the impact of ancestry-enriched SNPs on disease, metabolism and immune system pathways. This approach uncovered signals of ancestry-enrichment in health- and disease-related traits shared among all four of the Latin American populations that we analyzed, raising the possibility of shared selective pressures among them.

## Materials and methods

### Comparative genomic data sources

**Fig. 1:**
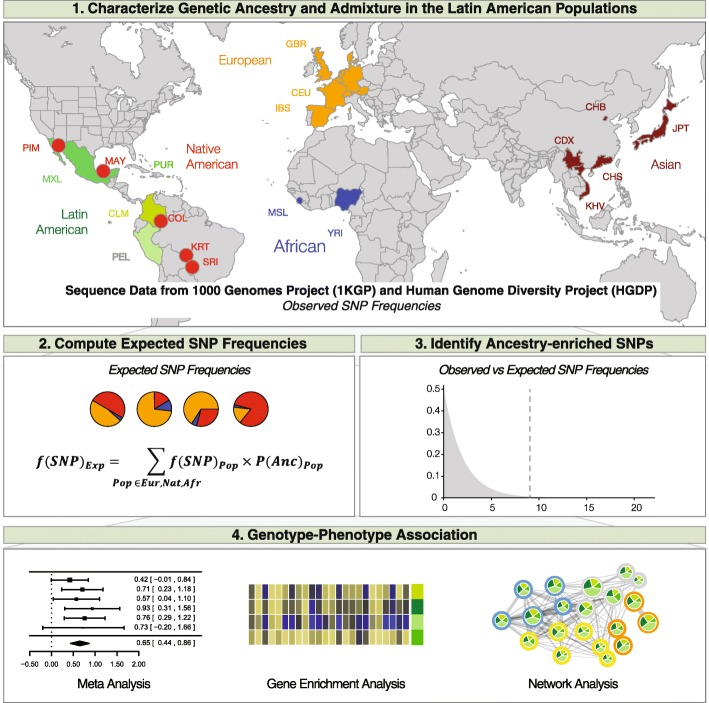
Analysis scheme used for this study. (1) Genetic ancestry and admixture profiles were characterized for four Latin American populations. (2) Expected SNP frequencies in the admixed Latin American populations are calculated based on their ancestry profiles. (3) Ancestry-enriched SNPs are identified by comparing observed versus expected SNP allele frequencies in in the admixed Latin American populations. (4) Ancestry-enriched SNPs are mapped to genes, which in turn are used for gene set enrichment in order to identify impacted health-related pathways and phenotypes

Whole genome sequences from four admixed Latin American populations – Colombia (*n* = 94), Mexico (*n* = 64), Peru (*n* = 85), and Puerto Rico (*n* = 104) – were taken from the 1000 Genomes Project (1KGP) phase 3 data release [19]: http://www.internationalgenome.org/data/. Genome sequences from the four Latin American populations were compared to whole genome sequences and whole genome genotypes of global reference populations from African, European, and Native American continental population groups to characterize their patterns of genetic ancestry and admixture (Fig. 1 and Table 1). The global reference whole genome sequences were also taken from the 1KGP, and reference whole genome genotype data was taken from the Human Genome Diversity Project (HGDP) [4]. The whole genome sequence and whole genome genotype data were merged, with sites that existed in both datasets retained for subsequent analysis, and PLINK v1.9 [20] was used to correct single nucleotide polymorphism (SNP) strand orientation as needed. This resulted in a dataset of 435,782 SNPs from 1449 individuals, across 19 populations. The final merged SNP dataset was phased with the 1KGP haplotype reference panel using the program SHAPEIT2 [21].

**Table 1:** Human populations analyzed in this study

Dataset	Short	Full Description	n
1KGP African (*n* = 193)	MSL	Mende in Sierra Leone	85
	YRI	Yoruba in Ibadan, Nigeria	108
1KGP East Asian (*n* = 504)	CDX	Chinese Dai in Xishuangbanna, China	93
	CHB	Han Chinese in Bejing, China	103
	CHS	Southern Han Chinese, China	105
	JPT	Japanese in Tokyo, Japan	104
	KHV	Kinh in Ho Chi Minh City, Vietnam	99
1KGP European (*n* = 297)	CEU	Utah residents with NW European ancestry	99
	IBS	Iberian populations in Spain	107
	GBR	British in England and Scotland	91
1KGP Admixed American (*n* = 347)	CLM	Colombian in Medellin, Colombia	94
	MXL	Mexican Ancestry in Los Angeles, California	64
	PEL	Peruvian in Lima, Peru	85
	PUR	Puerto Rican in Puerto Rico	104
HGDP Native American (*n* = 108)	KRT	Karitiana in Brazil	24
	SRI	Surui in Brazil	21
	COL	Colombians in Colombia	13
	MAY	Maya in Mexico	25
	PIM	Pima in Mexico	25

Populations are organized into continental groups: African, East Asian, European, and Admixed American from the 1000 Genomes Project (1KGP) and Native American from the Human Genome Diversity Project (HGDP). Short names, descriptions, and the numbers of genomes analyzed are provided for each individual population

### Genome ancestry assignment

ADMIXTURE [22] was run on individuals from both the global reference and admixed Latin American populations to infer their genome-wide ancestry profiles. ADMIXTURE was run with *K* = 3 corresponding the three ancestral continental population groups: African, European, and Native American. The ADMIXTURE results for the admixed Latin American populations were used to infer individuals’ percent ancestry contributions from each of these three continental ancestry groups. The program RFMix [23] was used to assign the continental ancestry origins of individual haplotypes across the genome, i.e. local ancestry. As with ADMIXTURE, African, European, and Native American populations were used as reference populations for RFMix. Ancestry-specific haplotypes were only called for regions where RFMix certainty was at least 99%.

### Detection of ancestry-enriched SNPs

Ancestry-enriched SNPs were characterized as SNPs found in higher frequencies in admixed Latin American populations compared to what is expected based on (1) their frequencies in the ancestral source populations, and (2) the proportion of ancestry derived from each ancestral source population. For any given SNP, in any given Latin American population, the expected frequency of the SNP *f*(*SNP*)_*Exp*_ can be calculated as:

1 $$ f{(SNP)}_{Exp}={\sum}_{Pop\in Eur, Afr, Nat}f{(SNP)}_{Pop}\times P{(Anc)}_{Pop} $$

where *f*(*SNP*)_*Pop*_ is the frequency of the SNP in a specific ancestral source population and *P*(*Anc*)_*Pop*_ is the proportion of ancestry in the modern Latin American population derived from that same ancestral population. Ancestry proportions were computed using the reduced, merged set of SNPs described above, with African and European reference populations from the 1KGP and Native American reference populations from HGDP. SNP frequencies were computed using whole genome sequences, in order to provide more complete coverage of variants genome-wide, using the YRI African and IBS European reference populations with the most closely related East Asian population CHB taken as a surrogate for Native American ancestry.

The statistical significance of SNP ancestry-enrichment calculated this way was determined by comparing the observed (*Obs*) to expected (*Exp*) frequencies of the reference (*Ref*) and alternate (*Alt*) alleles for any given SNP as shown here:

2 $$ {\chi}^2=\frac{{\left({Obs}_{Ref}-{Exp}_{Ref}\right)}^2}{Exp_{Ref}}+\frac{{\left({Obs}_{Alt}-{Exp}_{Alt}\right)}^2}{Exp_{Alt}} $$

The *χ*^2^ distribution was used to calculate *P*-values for each SNP, and false discovery rate (FDR) *q*-values were used to account for multiple statistical tests. The SNPs that had significant FDR values (*q* < 0.05) were considered to be ancestry-enriched in the Latin American population, i.e. present at a higher frequency than expected based on the population ancestry profile.

For ancestry-enriched SNPs, the individual ancestry components (*Anc*) that gave rise to the pattern of enrichment were also determined by jointly minimizing the frequency difference between the SNP in the Latin American population and a single ancestral source population while maximizing the distance between that single source population and the other two ancestral populations:

3 $$ {Anc}_{Pop1}=\left[\left(f{(SNP)}_{Pop1}-f{(SNP)}_{Pop2}\right)+\left(f{(SNP)}_{Pop1}-f{(SNP)}_{Pop3}\right)\right]/2-\left|f{(SNP)}_{Obs}-f{(SNP)}_{Pop1}\right| $$

where *f*(*SNP*)_*Popx*_ is the frequency of the SNP in each of the ancestral source populations and *f*(*SNP*)_*Obs*_ is the frequency of the ancestry-enriched SNP in the Latin American population.

### Gene set enrichment analysis

Ancestry-enriched SNPs were mapped to genes if they mapped within the NCBI RefSeq [24] gene models, i.e. between transcription start and stop sites, on the UCSC Genome Browser human genome reference sequence build GRCh37/hg19. Functionally coherent gene sets were curated from the Molecular Signatures Database (MSigDB) version 5.1 [25] for three broad functional categories: health- and disease-related phenotypes, metabolism, and immunity. Gene set enrichment analysis (GSEA) was performed for each Latin American population by adopting the MSigDB statistical framework to find functional gene sets that were enriched for genes with mapped ancestry-enriched SNPs. To do this, genes that harbor ancestry-enriched SNPs were overlapped with genes from each functional gene set, and overlap enrichment was performed using the R limma package [26]. Overlap enrichment *P*-values were computed for each gene set using the Wilcoxon signed-rank test.

### Expression quantitative trait loci (eQTL) analysis

RNA-seq data were taken from the GUEVADIS RNA sequencing (RNA-seq) project for 1KGP samples ftp://ftp.ebi.ac.uk/pub/databases/microarray/data/experiment/GEUV/E-GEUV-1/analysis_results/. A total of 445 RNA-seq samples were used in the analysis, including 87 African and 358 European individuals. The RNA-seq data correspond to gene expression levels for the same lymphoblastoid cell lines, i.e. Epstein–Barr virus (EBV) transformed B-lymphocytes, which were used for the 1KGP DNA-seq characterization. RNA-seq sample preparation, sequencing experiments and read-to-genome mapping were performed as previously described [27]. The read-to-genome mapping corresponds to human genome build GRCh37/hg19. Gene expression levels were quantified based on RNA-seq mapped reads and corresponded to ENSEMBL gene models [28]. Gene expression levels were quantified by using the reads per kilobase per million mapped reads (RPKM) approach in combination with the probabilistic estimation of expression residuals (PEER) method as previously described [29, 30].

Matched whole genome sequencing based SNP genotype calls were obtained for the same 445 individuals from the phase 3 release of the 1KGP [19]. Only SNPs with minor allele frequency (MAF) greater than 5% were used for the downstream analysis to ensure both the confidence of genotype calls and the reliability of the eQTL association analyses. Gene expression levels of 445 individuals were then regressed against their SNP genotypes to identify eQTLs using the program Matrix eQTL [31]. The Matrix eQTL program was run using the additive linear model option with gender and population labels included as covariates.

### SNP pathway meta-analysis

Meta-analysis was used to evaluate the overall ancestry-enrichment for sets of SNPs that are implicated in specific health- or disease-related pathways. For any given ancestry-enriched SNP that mapped to a gene found in an overrepresented pathway, a log odds ratio was calculated as:

4 $$ Log\  Odds\ Ratio=\mathit{\ln}\left[\frac{f{(Obs)}_{Ref}/f{(Exp)}_{Ref}}{f{(Obs)}_{Alt}/f{(Exp)}_{Alt}}\right] $$

where the observed (*Obs*) versus expected (*Exp*) frequencies (*f*) are compared for the SNP reference (*Ref*) versus alternate (*Alt*) alleles. Then for each pathway, the set of individual SNP log odds ratios was combined to yield pathway-specific log odds ratio values along with 95% confidence intervals using the fixed-effect model with moderators via linear (mixed-effect) models implemented in the metafor package in R [32].

## Results

### Relating genome ancestry and health in Latin America

We developed and applied a single nucleotide polymorphism (SNP)-based approach to relate genome ancestry to genetic determinants of health in admixed Latin American populations (Fig. 1). First, patterns of genetic ancestry and admixture in Latin American populations were characterized via comparison with reference genome sequences of putative ancestral source populations from Africa, the Americas and Europe (Table 1). We then computed the expected SNP frequencies in Latin American populations by taking into consideration the SNP frequencies in the ancestral source populations along with the proportional contributions of each ancestral source population to the modern Latin American populations. Comparisons of observed versus expected SNP frequencies in admixed Latin American populations were used to identify what we refer to as ‘ancestry-enriched’ SNPs, which are SNPs found at anomalous frequencies in Latin American populations compared to what can be expected based on their ancestry profiles. Ancestry-enriched SNPs were mapped to genes, and then genes were used in gene set enrichment analysis to identify impacted health-related pathways and phenotypes.

### Genetic ancestry and admixture in four Latin American populations

**Fig. 2:**
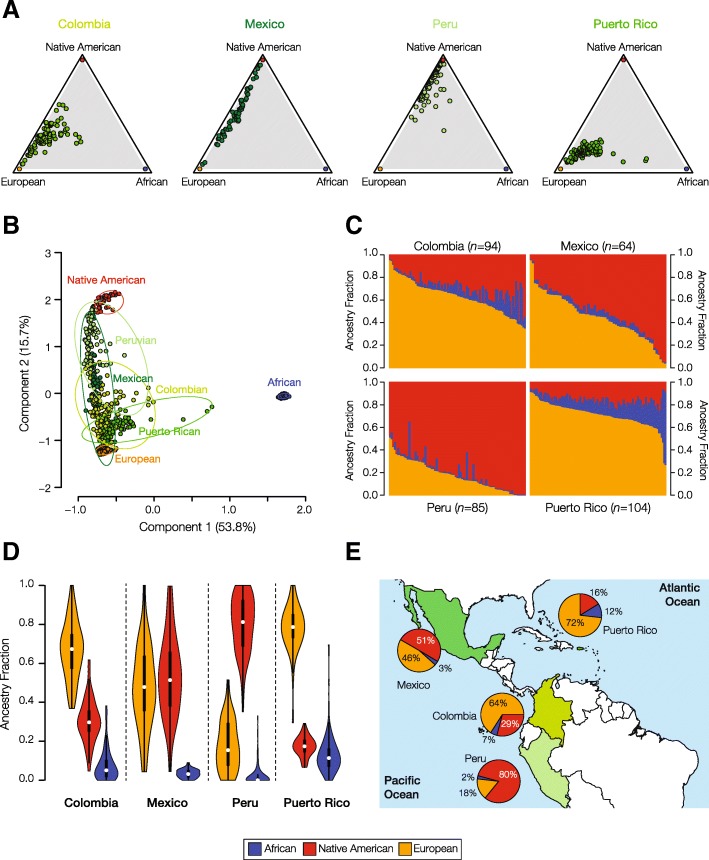
Genetic ancestry and admixture in Latin American populations. The ancestry contributions of putative ancestral source populations to four modern, admixed Latin American populations are shown. **a** Triangle plots showing the relative ancestry contributions – African, European, Native American – to admixed individuals from four Latin American populations. **b** PCA plot showing the genetic relationships among individuals from admixed Latin American populations compared to putative ancestral source populations. Each population is bounded by a minimum spanning ellipse. **c** Admixture plots showing the fractions of African, Native American and European ancestry among admixed individuals from four Latin American populations. Each individual is represented as a column with the admixture fractions color coded as shown in the legend. **d** Violin plots showing distributions of ancestry fractions among individuals from four Latin American populations. **e** Pie charts showing the average ancestry values for each population next to its geographic location

Genome sequences from four Latin American populations – Colombia, Mexico, Peru, and Puerto Rico – were compared to whole genome sequences and whole genome genotypes of global reference populations from African, European, and Native American continental population groups in order to characterize their patterns of genetic ancestry and admixture. Each Latin American population has a distinct pattern of three-way continental genetic admixture characterized by population-specific proportions of African, European and Native American ancestry (Fig. 2). Puerto Rico and Colombia are characterized by relatively high levels of three-way admixture, with substantial ancestry contributions from all three continental population groups, whereas Mexico and Peru have primarily Native American and European ancestry. Puerto Rico and Colombia also have the highest levels of European ancestry, while Peru and Mexico have majority Native American ancestry. The 80% Native American ancestry component for Peru is the single highest contribution of any ancestral population to an admixed Latin American population, and the 2% African ancestry fraction for this same population is the lowest. African source populations contribute the least amount of ancestry to all four Latin American populations analyzed here. The continental ancestry proportions for each Latin American population were used as described in the following section to detect ancestry-enriched SNPs that exist in any given population at a higher frequency than expected based on its ancestry profile.

### Ancestry-enriched SNPs in Latin American populations

Our approach to relating genetic ancestry to determinants of health and disease in modern Latin American populations relies on the detection of SNPs that are found at anomalously high frequencies in admixed populations compared to what is expected based on their frequencies in the ancestral source populations, i.e. ancestry-enriched SNPs. We reasoned that such ancestry-enriched SNPs are likely to have an outsized effect on health and disease in modern Latin American populations, perhaps related to an initial increase in population frequency via adaptive introgression.

We developed and applied a quantitative method to identify individual SNPs that are enriched in admixed Latin American populations with respect to ancestry from one of the three ancestral source populations: Africa, Europe, and the Americas. To do so, the expected frequencies for each SNP were calculated using the frequency of the given SNP in each of the three ancestral source populations conditioned upon the proportion of each ancestral source population in the Latin American population of interest. Observed SNP frequencies were compared to expected SNP frequencies to identify ancestry-enriched SNPs; the details of this approach are shown in the Materials and Methods section.

**Fig. 3:**
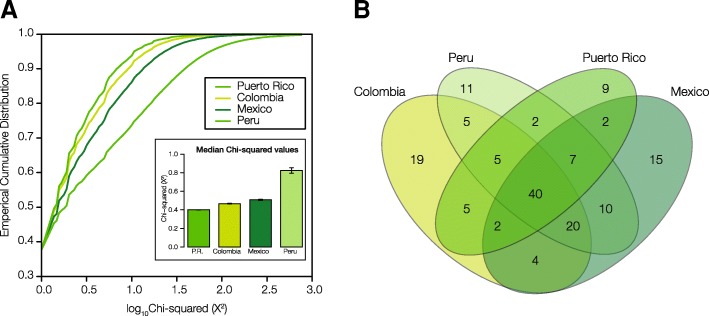
Ancestry-enriched SNPs in Latin American populations. An overview of the distributions of ancestry-enriched SNPs within and between the four admixed Latin American populations are shown, giving an indication of the overall numbers of ancestry-enriched SNPs along with the extent to which they are shared or unique to specific populations. **a** Cumulative distributions of ancestry-enrichment *χ*^2^ values for all SNPs in the four Latin American populations. Inset: Median *χ*^2^ values for each population ± standard error. **b** Venn diagram showing the number of genes with significant ancestry-enriched SNPs exclusive to one population and those shared by more than one population

The distributions and median values of ancestry-specific SNP χ^2^ values are shown in Fig. 3a. Peru shows the strongest overall signal of SNP ancestry-enrichment, followed by Mexico, Colombia, and Puerto Rico. Statistically significant ancestry-enriched SNPs for each population were mapped to genes for subsequent analysis of their impact on health- and disease-related phenotypes. There is a substantial amount of overlap of mapped genes among the four populations (Fig. 3b). Out of 156 total genes with mapped ancestry-enriched SNPs, 102 (65%) are shared among two or more populations compared to 54 (35%) that are population-specific. There are 40 genes that bear ancestry-specific SNPs in all four Latin American populations, which is by far the single largest component of shared versus unique genes. Lists of all SNPs that show significant ancestry-enrichment – along with details regarding their observed and expected allele frequencies, test-statistic values, and specific ancestry enrichment patterns – can be found in Additional file 1: Table S1.

### Gene set enrichment analysis of overrepresented SNPs

**Fig. 4:**
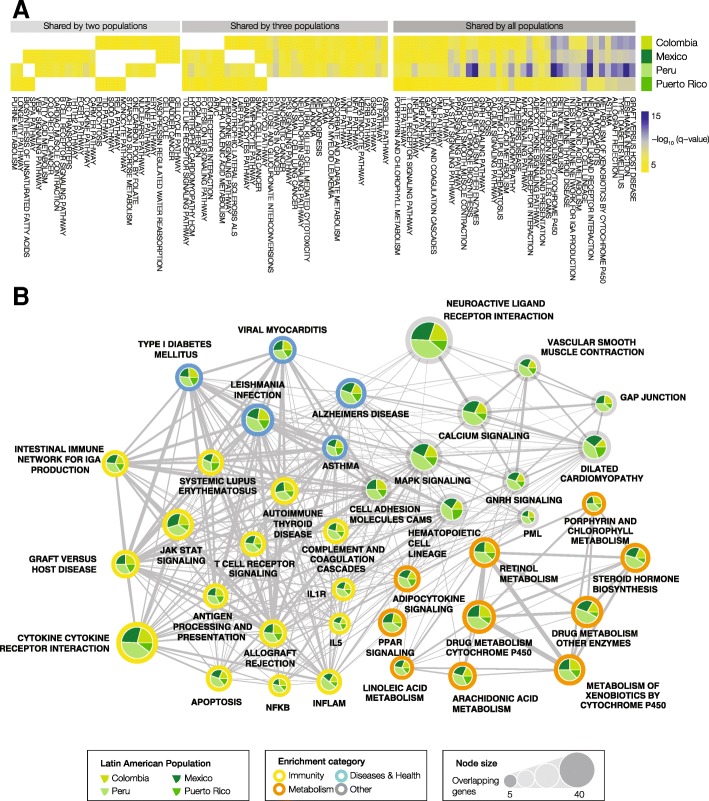
Gene set enrichment analysis of ancestry-enriched SNP genes. Functionally coherent gene sets and pathways that are overrepresented with respect to ancestry-enriched SNPs are shown, giving an indication of the kinds of health-related phenotypes that have been shaped by genetic ancestry in the four admixed Latin American populations. **a** Heatmap showing significantly enriched functional gene sets (i.e., pathways and phenotypes) shared by two, three or all four Latin American populations. The cells are color coded by the log transformed statistical significance (FDR *q*-value) of gene set enrichment analysis results. **b** Network showing significantly enriched pathways and phenotypes shared by all four Latin American populations. Nodes sizes represent the number of ancestry-enriched genes in each set. Pie charts show how many genes in a given set are from each population. Color coding describes the functional enrichment category as shown in the legend

The genes with mapped ancestry-specific SNPs were analyzed with gene set enrichment analysis (GSEA) to look for overrepresented health- or disease-related pathways and phenotypes (Fig. 4). This approach allowed us to identify the specific pathways and phenotypes that are most affected by ancestry-enriched SNPs. The presence of significantly overrepresented pathways and/or phenotypes in two or more populations was taken to indicate a higher likelihood of genetic ancestry effects on health and disease in modern Latin American populations.

A number of pathways and phenotypes have significantly overrepresented ancestry-enriched SNP genes in all four populations (Fig. 4a). These include gene sets related to immunity (yellow) and metabolism (orange) as well as several disease-specific gene sets (blue) (Fig. 4b). Immune system pathways with ancestry-enriched SNPs include the cytokine receptor interaction, T cell receptor signaling, and antigen processing and presentation pathways. Implicated metabolic pathways include both drug and xenobiotic metabolism as well as steroid hormone biosynthesis. Diseases of note include several pathologies that are known to be found in high prevalence in Latin American populations: type I diabetes, Alzheimer’s disease and Leishmaniasis. A number of other signaling pathways were implicated by this analysis – calcium, MAPK, and GNRH signaling – pointing a role for ancestry-enriched SNPs in mediating human-environment interactions. Lists of all pathways that show significant enrichment of genes with mapped ancestry-enriched SNPs, for each admixed Latin American population – along with information regarding the overlapping genes and pathway enrichment statistical significance (FDR *q*-values) – are provided in Additional file 2: Table S2.

**Fig. 5:**
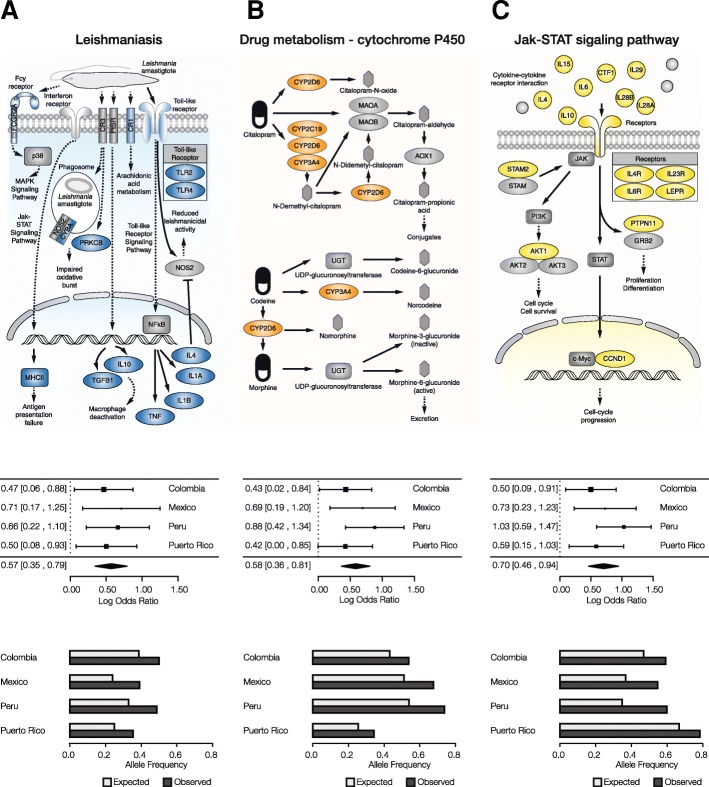
Pathways with ancestry-enriched SNP genes in functional categories of interest**.** These results highlight examples of specific health-related functions and pathways that have been shaped by genetic ancestry in the four admixed Latin American populations. For each functional category, a pathway schematic is shown, indicating the pathway genes and their roles, along with meta-analysis results and observed versus expected SNP frequencies for each population. **a** Leishmaniasis, an example of a disease and health related pathway. **b** Cytochrome P450 drug metabolism, an example of a metabolism related pathway. **c** Jak-STAT signaling pathway, an example of an immune-related pathway

We focused on several notable examples of health- and disease-related pathways that were found to have significantly overrepresented ancestry-enriched SNP genes in all four Latin American populations (Fig. 5). For each of these pathways, and in each population studied, we performed additional meta-analyses of the sets of mapped ancestry-enriched SNPs in order to evaluate the pathway’s overall ancestry enrichment. We also computed analogous overall observed versus expected allele frequencies for each pathway in all four populations. There are 15 genes from the Leishmaniasis immune response pathway with mapped ancestry-enriched SNPs, including a pair of Toll-like Receptor encoding genes as well as several interleukin genes (Fig. 5a). The meta-analysis for this pathway shows an overall ancestry-enrichment for all SNPs in each of the four populations analyzed here. Leishmaniasis is a parasitic disease with high prevalence in the tropics and subtropics including Latin America. Similar pathway-specific analysis revealed overall ancestry-enrichment for SNPs linked to drug metabolism (Fig. 5b), including multiple genes from the cytochrome P450 family, as well as the Jak-STAT signaling pathway, which is activated by cytokines as part of the innate immune response (Fig. 5c). The ancestry-enrichment observed for the drug metabolism pathway could represent an adaptation based on detoxification linked to local dimensions of diet and environmental exposure in the New World.

### Ancestry-specific expression quantitative trait loci (eQTL)

We explored the effects of ancestry-specific SNPs on gene regulation via expression quantitative trait loci (eQTL) analysis. eQTL are individual SNPs with genotype variants that are associated with gene expression levels; associations of this kind point to a role for SNP variants in gene regulation (e.g., via differential transcription factor binding affinities and/or allele specific expression levels) [33, 34]. To do this, we searched for ancestry-enriched SNPs that have ancestry-specific or shared genotype-expression associations. The first step of this analysis entailed the identification of the specific ancestry-components that predominantly contribute to the observed patterns of SNP ancestry-enrichment (see Materials and Methods). SNPs with highly asymmetric ancestry-enrichment patterns, i.e. predominant contributions from a single ancestral source population, were then chosen for eQTL analysis.

**Fig. 6:**
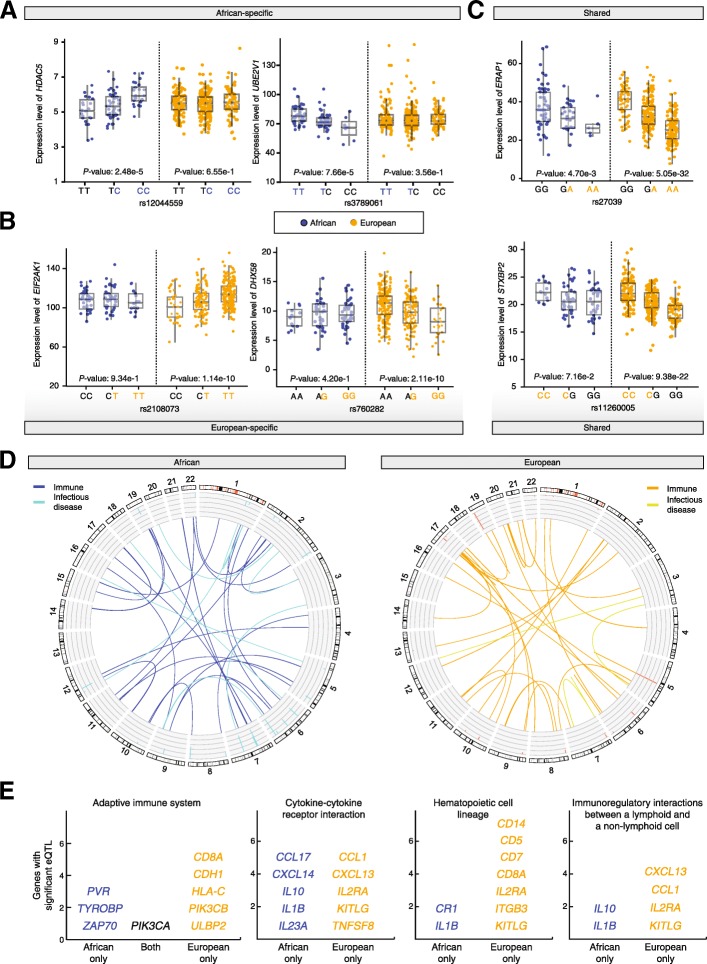
Ancestry-specific effects on gene expression. These results give an indication of how ancestry-enriched SNPs can impact health-related phenotypes by virtue of their gene regulatory effects. SNP-by-ancestry interactions were characterized using an expression quantitative trait loci (eQTL) approach. Examples of **a** African-specific and **b** European-specific eQTL are shown along with **c** eQTL shared between populations. **d** eQTL related to immune system and infectious disease found in the African and European populations are shown in a CIRCOS plot with links indicated between eQTLs and their regulated genes. **e** Examples of immune-related pathways that include multiple eQTL-regulated genes for African and/or European populations

Using this approach, we found a number of cases of SNPs that show overrepresented African or European ancestry in modern Latin American populations and are also associated with ancestry-specific gene regulation (Fig. 6). A number of the genes regulated by ancestry-specific SNPs were found to play specific roles in the immune system and infectious disease responses. In particular, genes from both the innate and adaptive immune system were found to be regulated by ancestry-enriched SNPs that exert population-specific regulatory effects (Fig. 6a-d). For example, African ancestry-enriched SNPs were found to exert African-specific regulatory control over genes for both immunoglobulin receptors (PVR and TYROBP) and a downstream tyrosine kinase (ZAP70) involved in the adaptive immune response (Fig. 6e). Similarly, European ancestry-enriched SNPs were also found to act as population-specific eQTLs with regulatory effects on that were specific to the European populations. Analogous patterns of ancestry-specific SNP enrichment and gene regulatory control were found for genes involved in cytokine-receptor interactions, hematopoietic cell development, and cell-cell immunomodulatory interactions.

## Discussion

Latin America has a unique genetic heritage with high levels of admixture from African, European, and Native American ancestral source populations [9–11]. As such, the genome sequences of Latinos contain combinations of ancestry-specific genetic variants that never previously existed in the same genomic background. In other words, Latin American genomes represent a very recent evolutionary innovation in the long trajectory of human evolution and migration around the globe. Accordingly, the development and application of genomic approaches to healthcare in Latin America will require a deep understanding of the genetic ancestry and admixture profiles of Latin American populations. This issue is particularly pressing given the fact that the vast majority of studies aimed at uncovering genetic variants associated with health- and disease-related phenotypes have been conducted in populations with European ancestry [35, 36].

Here, we have tried to address this issue by relating patterns of genetic ancestry and admixture to health and disease determinants in Latin American genomes. To do so, we developed and applied a novel SNP-based approach to ancestry enrichment analysis. Our approach leverages information on the genetic ancestry of the modern Latin American populations to discover SNP variants that exist in a given population at higher frequencies than expected, i.e. ancestry-enriched SNPs. We found that specific sets of ancestry-enriched genetic variants, from each of the three ancestral source populations, have been preferentially retained in modern Latin American populations based on a variety of roles that they play in health and fitness. These findings have relevance for the development of genomic approaches to healthcare, i.e. personalized or precision medicine, in Latin America.

Gene set enrichment analysis uncovered a number of immunity, metabolism, and disease-related pathways that are significantly overrepresented with respect to genes that contain ancestry-enriched SNPs (Figs. 4 and 5). These results suggest that these particular pathways, and their related phenotypes, could underlie population-specific health disparities in the four admixed Latin American populations studied here. They also give an indication that populations with particular ancestry profiles may be more or less disposed to some of these diseases and phenotypes; information of this kind could ultimately help to guide targeted health interventions. As these results represent basic research into the relationship between genetic ancestry and determinants of health, more clinically facing (translational) research will need to be done in order to precisely define the role of individual ancestry-enriched variants in disease etiology, prevention and treatment.

Expression quantitative trait loci (eQTL) analysis revealed ancestry-enriched SNPs in modern Latin American populations that are associated with African- or European-specific patterns of gene regulation (Fig. 6). This includes SNPs that are associated with ancestry-specific regulation of genes involved in both the innate and adaptive immune systems as well as targeted infectious disease responses. These results underscore the relevance of gene regulatory control as an underlying driver of adaptive introgression in admixed populations.

One important caveat with respect to the interpretation of the results that we report is that they can only be taken to apply to the four specific populations analyzed here: Colombian in Medellin, Colombia (CLM), Mexican Ancestry in Los Angeles, California (MXL), Peruvian in Lima, Peru (PEL), and Puerto Rican in Puerto Rico (PUR). Given the diversity of Latin American populations, and in particular their distinct ancestry profiles, we should expect to see distinct ancestry enrichments for different countries in the region, such as Argentina, Chile, Brazil etc. This caveat not only applies to different countries but also applies to different populations within the same country. Colombia, for instance, is an extremely diverse country with populations from different regions that show very distinct ancestry profiles [9]. The population of Colombia analyzed here is from Medellín in the state of Antioquia, and this particular population shows averages of 64% European ancestry, 29% Native American ancestry, and 7% African ancestry. However, we have previously shown that the population from the neighboring state of Chocó has a totally distinct ancestry profile with 76% African ancestry, 13% European ancestry, and 11% Native American ancestry [37–39]. Accordingly, results from the analysis of the population from Medellín cannot be taken to represent the entire country of Colombia. Clearly, a deeper understanding of the relationship between genetic ancestry and health determinants in Latin America will require analysis of many more populations within and between the region’s countries.

## Additional files

Additional file 1: **Table S1.** Lists of all SNPs that show significant ancestry-enrichment. For each admixed Latin American population, all ancestry-enriched SNPs (*q* < 0.05) are given along with their meta-information, observed and expected reference and alternate allele frequencies, and significance values. The allele (reference or alternate) and enriched ancestry (African, European or Native American) are designated for each ancestry-enriched SNP. (XLSX 177 kb)

Additional file 2: **Table S2.** Lists of pathways that show significant enrichment of genes with mapped ancestry-enriched SNPs for each admixed Latin American population. For each KEGG pathway with significant enrichment of genes with mapped ancestry-enriched SNPs in at least one of the four populations, the overlapping genes and FDR *q*-values are given. (XLSX 29.6 kb)

## Abbreviations

1KGP: 1000 genomes project
DNA-seq: DNA sequencing
eQTL: Expression quantitative trait locus
GEUVEDIS: Genetic European Variation in Health and Disease
HGDP: Human Genome Diversity Project
RNA-seq: RNA sequencing
SNP: Single nucleotide polymorphism
